# Novel Ampeloviruses Infecting Cassava in Central Africa and the South-West Indian Ocean Islands

**DOI:** 10.3390/v13061030

**Published:** 2021-05-29

**Authors:** Yves Kwibuka, Espoir Bisimwa, Arnaud G. Blouin, Claude Bragard, Thierry Candresse, Chantal Faure, Denis Filloux, Jean-Michel Lett, François Maclot, Armelle Marais, Santatra Ravelomanantsoa, Sara Shakir, Hervé Vanderschuren, Sébastien Massart

**Affiliations:** 1 Plant Pathology Laboratory, TERRA-Gembloux Agro-Bio Tech, University of Liège, Passage des Déportés, 2, 5030 Gembloux, Belgium; arnaud.blouin@uliege.be (A.G.B.); Francois.maclot@chuliege.be (F.M.); 2 Faculté des Sciences Agronomiques, Université Catholique de Bukavu, BP 285 Bukavu, Democratic Republic of the Congo; ebisimwa@yahoo.com; 3 Earth and Life Institute, Applied Microbiology-Phytopathology, UCLouvain, 1348 Louvain-la-Neuve, Belgium; claude.bragard@uclouvain.be; 4 Université Bordeaux, INRAE, UMR BFP, CS20032, CEDEX, 33882 Villenave d’Ornon, France; thierry.candresse@inrae.fr (T.C.); chantal.faure@inrae.fr (C.F.); armelle.marais-colombel@inrae.fr (A.M.); 5 CIRAD, UMR PHIM, 34090 Montpellier, France; denis.filloux@cirad.fr; 6 PHIM Plant Health Institute, Université Montpellier, CIRAD, INRAE, Institut Agro, IRD, 34000 Montpellier, France; 7 CIRAD, UMR PVBMT, Pôle de Protection des Plantes, Saint-Pierre, F-97410 Ile de la Reunion, France; jean-michel.lett@cirad.fr; 8 FOFIFA-CENRADERU, Laboratoire de Pathologie Végétale, BP 1444 Ambatobe, Madagascar; srakotoarisoaravel@gmail.com; 9 Plant Genetics Laboratory, TERRA-Gembloux Agro-Bio Tech, University of Liège, Passage des Déportés, 2, 5030 Gembloux, Belgium; shakir.sara@yahoo.com (S.S.); herve.vanderschuren@uliege.be (H.V.); 10 Laboratory of Tropical Crop Improvement, Division of Crop Biotechnics, Biosystems Department, KU Leuven, 3000 Leuven, Belgium

**Keywords:** *Manihot esculenta*, *Ampelovirus*, high-throughput sequencing, Central Africa, Indian Ocean islands, *Closteroviridae*

## Abstract

Cassava is one of the most important staple crops in Africa and its production is seriously damaged by viral diseases. In this study, we identify for the first time and characterize the genome organization of novel ampeloviruses infecting cassava plants in diverse geographical locations using three high-throughput sequencing protocols [Virion-Associated Nucleotide Acid (VANA), dsRNA and total RNA], and we provide a first analysis of the diversity of these agents and of the evolutionary forces acting on them. Thirteen new *Closteroviridae* isolates were characterized in field-grown cassava plants from the Democratic Republic of Congo (DR Congo), Madagascar, Mayotte, and Reunion islands. The analysis of the sequences of the corresponding contigs (ranging between 10,417 and 13,752 nucleotides in length) revealed seven open reading frames. The replication-associated polyproteins have three expected functional domains: methyltransferase, helicase, and RNA-dependent RNA polymerase (*RdRp*). Additional open reading frames code for a small transmembrane protein, a heat-shock protein 70 homolog (*HSP70h*), a heat shock protein 90 homolog (*HSP90h*), and a major and a minor coat protein (*CP* and *CPd* respectively). Defective genomic variants were also identified in some cassava accessions originating from Madagascar and Reunion. The isolates were found to belong to two species tentatively named Manihot esculenta-associated virus 1 and 2 (MEaV-1 and MEaV-2). Phylogenetic analyses showed that MEaV-1 and MEaV-2 belong to the genus *Ampelovirus*, in particular to its subgroup II. MEaV-1 was found in all of the countries of study, while MEaV-2 was only detected in Madagascar and Mayotte. Recombination analysis provided evidence of intraspecies recombination occurring between the isolates from Madagascar and Mayotte. No clear association with visual symptoms in the cassava host could be identified.

## 1. Introduction

Approximately 40% of the world’s area dedicated to the cultivation of roots and tubers is planted with cassava. In Africa, 56% of the total production of roots and tubers comes from cassava, 51% of which are from Nigeria and the Democratic Republic of Congo (DR Congo) [1]. Cassava is one of the most important staple crops, as it ranks fourth as a source of calories for human consumption [1]. Many of the poorest farmers and most undernourished households in Africa depend on cassava as a principal nutrition source. It also constitutes an important income source in rural and marginal areas and has multiple uses, most notably as a food security and regular food crop [2].

Due to its remarkable adaptability to a wide range of soil and environmental conditions, cassava is bestowed with resilience to global warming and climate change, and with a potential for better return under adverse soil and weather conditions [3]. The exceptional soil carbon sequestration properties of cassava makes it a potential crop for improving the green revolution fatigue [4].

Despite the large contribution of Africa to global cassava production (60%), its performance in terms of yield is the lowest (9 tons/ha on average) [1]. In most African cassava-producing areas, the yield is far below the potential [5]. In DR Congo, yields of approximately 8 tons/ha can be obtained under farmers’ fields conditions, but yields are highly variable depending on the local pedo-climatic conditions.

Cassava suffers from many pests and diseases, including viruses, which can seriously affect the quality and quantity of the harvest as well as the quality of the planting materials. Two viral diseases are of major economic importance in sub-Saharan Africa, namely, cassava brown streak disease (CBSD) and cassava mosaic disease (CMD) [6]. CBSD is associated with two *Ipomovirus* species, collectively named cassava brown streak viruses (CBSVs), and CMD is associated with nine *Begomovirus* species, collectively named cassava mosaic geminiviruses (CMGs) [6]. Yield reduction due to CMD may be severe and losses up to 82% have been reported, especially in cassava plants dually infected with African cassava mosaic virus (ACMV) and the Ugandan strain of East African cassava mosaic virus (EACMV-UG) [7].

The family *Closteroviridae* is a large and diverse group of filamentous plant viruses (particles of 650−2200 nm in length) with single-stranded RNA genomes. Some species can be transmitted semi-persistently by aphids, whiteflies, mealybugs or soft scales insects [8,9]. The members of this family are known to affect several crops of major economic importance, such as sugar beet, citrus, tomato, lettuce, potato, sweet potato, grapevine, pineapple, cherry, and some ornamentals [9,10,11,12,13].

To date, 56 virus species have been classified as definitive or tentative members of the family *Closteroviridae* [14]. They are grouped into the following four genera: *Ampelovirus* (monopartite genome, mealybugs and soft scale insect vectors), *Closterovirus* (monopartite genome, aphid vectors), *Velarivirus* (monopartite genome, no known vectors) and *Crinivirus* (bipartite genome, whitefly vectors) [14,15].

The accepted demarcation criteria between the species in the *Closteroviridae* family [9,16] include the following aspects: (i) particle size, (ii) size of *CP*, as determined by the deduced amino acid sequence data, (iii) genome structure and organization (number and relative location of the open reading frames (ORFs)), (iv) amino acid sequence of the relevant gene products (*CP, RdRp, HSP70h*), differing by more than 25%, (v) vector species and specificity, (vi) magnitude and specificity of the natural and experimental host range, and (vii) cytopathological features (aspect of inclusion bodies and origin of cytoplasmic vesicles) [14].

Specifically, the genus *Ampelovirus* comprises species with linear particles of 1400−2000 nm long. Their genome is a monopartite positive sense, single-stranded RNA of 13.7−18.5 kb with a number of ORFs varying between 7 and 12 [9]. The members of this genus are divided into two subgroups accommodating, respectively, seven species with large (15,000 to over 18,000 nt) and complex (9 to 12 ORFs) genomes (subgroup I), and five species with smaller (13,000−14,000 nt) and simpler (7 ORFs) genomes (subgroup II) [9,16].

The majority of the *Ampelovirus* species have been identified from woody hosts (grapevine, *Prunus* sp., figs) and pineapple. Although their pathogenicity sometimes remains unclear, several members are reported to induce a diverse range of symptoms, while others are reported to have no association with symptoms. Natural vectors are *Pseudococcidae* mealybugs and soft scale insects, which transmit viruses in a semipersistent manner [9,12]. None of the known ampeloviruses are transmitted through seeds or mechanically. All ampeloviruses persist in plant parts used for vegetative propagation and are disseminated with them over long distances. The geographical distribution is therefore usually wide [9].

In this study, we identify for the first time and characterize the genome organization of novel ampeloviruses infecting cassava plants originating from diverse geographical locations in central Africa and the southwestern Indian Ocean islands. We also provide a first analysis of the diversity of these agents and of the evolutionary forces acting on them.

## 2. Materials and Methods

### 2.1. Origin of the Analyzed Cassava Samples and High-Throughput Sequencing

Field surveys were conducted from March to May 2016 in the South Kivu province (DR Congo). Cuttings were collected on cassava landraces plants showing foliar CBSD-like symptoms. These cuttings did not express the foliar symptoms anymore after growing in the greenhouse for six months. In addition, leaf samples from plants of different landraces were collected from the germplasm collections of the Conseil Départemental (DARTM in Mayotte), of CIRAD (Reunion) and of FOFIFA (Madagascar) between 2015 and 2017 (Table 1).

For the two samples from DR Congo, total RNA was extracted from cassava leaves using the RNeasy Plus Plant Mini Kit (QIAGEN^®^, Hilden, Germany) according to the manufacturer’s instruction and quality tested using a Nanodrop 2000 spectrophotometer (Thermo Fisher Scientific, Waltham, MA, USA). DNase treatment was applied using Amplification grade DNASE I (Life Technologies, California, USA) according to the manufacturer’s instructions. Ribosomal RNAs were depleted using a RiboZero plant leaf kit for RNA-seq (Life Technologies Limited, Paisley, UK), and libraries were prepared following the manufacturer’s instructions using a TrueSeq stranded total RNA kit (Illumina, New York, NY, USA). The RNA libraries were sequenced on a Nextseq 500 sequencing machine at the University of Liege (Liege, Belgium), with a read length of 2 × 75 nt.

For the three samples from Mayotte and Reunion, double-stranded RNAs (dsRNAs) were extracted from leaf samples using the procedure of Marais et al. [17]. Purified dsRNAs were converted to cDNA and amplified using a random whole-genome amplification procedure [17] and finally sequenced using Illumina Myseq technology (2 × 250 nt paired reads) on the Genotoul INRAE platform (Toulouse, France).

For the two samples from Madagascar, HTS was performed using a virion-associated nucleic acid (VANA)-based metagenomics approach as described by Palanga et al. [18].

### 2.2. Bioinformatic Analyses

Samples from Madagascar, Mayotte and Reunion: Following demultiplexing and quality trimming, reads were assembled into contig using CLC Genomic Workbench 8.5.1 and following versions. Contigs were annotated by BLASTx analysis against the GenBank protein database. Contigs were then manually assembled into scaffolds, which were consolidated and extended by several rounds of mapping of reads to yield the finalized scaffolds sequences. For two of the isolates (RE-Ljv and MY-Ren), reads corresponding to deletion events generating defective molecules were identified during this assembly process and scaffolds corresponding to these DI RNAs were therefore also reconstructed.

Samples from DR Congo: Sequence analyses were done using the Geneious 11.1.3 environment (www.geneious.com and embedded plugins). Data were submitted to a pre-processing step consisting of setting paired reads, and trimming adaptor sequences and low-quality reads (by BBDUK). Unique sequences were generated as clean reads by merging paired reads (by BBMerge) and removing duplicate reads (Dedupe). SPADES was used for de novo assembly. Reconstructed contigs were screened against the Refseq viral database retrieved from NCBI (12 October 2018) using BLASTn and BLASTx searches and annotated through tBLASTx. Viral contigs were further analyzed directly on the NCBI site using a BLASTn and BLASTx search with standard parameters.

Identification of functional gene domains for all samples was done by submitting predicted open reading frames (ORFs) directly on the NCBI’s conserved domain search tool (https://www.ncbi.nlm.nih.gov/Structure/cdd/wrpsb.cgi) (accessed from October to December 2018).

Multiple sequence alignments were built with ClustalW embedded in Mega X. Maximum likelihood phylogenetic trees were reconstructed in Mega X using the GTR+GI model for the alignment of nucleotide sequences and a Poisson model with uniform distribution for amino acid sequence alignments. Bootstrap analysis (100 replicates) was performed in order to evaluate the stability and significance of branches. Phylogenetic trees of amino acid sequences were reconstructed using only isolates for which the amino acid sequence of the analyzed protein was complete or nearly complete.

Putative recombination events were detected and evaluated using the RDP4 program (version 4.99) [19] and a ClustalW-built multiple alignment of the complete genome sequences.

### 2.3. Confirmatory RT-PCR and Sequencing

The presence of the detected viral contigs of MEaV-1 (isolates CG-Nmb and CG-Kah) in the corresponding plants was confirmed by RT-PCR using specific primer pairs (Table 2) designed according to the consensus sequence of the reconstructed viral contigs. This also allowed to confirm the sequence of the contigs of isolates mentioned.

### 2.4. Characterization of Defective RNA (D-RNA Molecules)

#### 2.4.1. RNA Isolation, cDNA Synthesis and Amplification of D-RNAs

Total RNA extracted from cassava accessions in which defective RNA molecules have been identified (RE-Ljv and MG-Mena-242) was purified and used for cDNA synthesis using the ProtoScript II First Strand cDNA Synthesis Kit (NEB) following the manufacturer’s instructions. Briefly, 0.5 μg of RNA was mixed with 2 μL of random hexamers primers (60 μM), heated at 65 °C for 5 min, and immediately cooled on ice for 5 min. The cooled solution was mixed with 10 μL of 2x ProtoScript II reaction mix and 2 μL of 10x ProtoScript II enzyme mix, mixed well and centrifuged for 5 s. This reaction mixture was incubated at 25 °C for 5 min and 42 °C for 1 h. The reaction was later terminated by incubation at 80 °C for 5 min.

Specific primer pairs designed on genome regions flanking the deletion in each of the four defective RNA molecules (Table 3) were used to amplify a region spanning the deletion of the D-RNAs. PCR amplification was performed in 20 μL total volume with 10 μL of Q5 High-Fidelity Master Mix (NEB), 2 μL of cDNA template, 0.5 μL of 10 μM of each primer and nuclease-free water for the remaining volume. The amplification program was set using 98 °C for 3 min of initial denaturation, followed by 35 cycles of 98 °C for 10 s, 57 °C for 10 s, 72 °C for 30 s and a final extension at 72 °C for 5 min. The amplified PCR products were analyzed on 1% agarose gel to validate the presence of amplicons of the expected size. The amplicons were gel-purified using the Monarch DNA Gel Extraction Kit (NEB) and cloned into pJET1.2 (Thermo Fisher Scientific).

#### 2.4.2. Sequence Analysis and Alignments

The amplicons clones were Sanger-sequenced using pJET1.2 forward and reverse primers. Two clones were sequenced for each defective RNA molecule and no sequence variation was observed among them. The obtained sequences were aligned to reference parental full-length segments (RE-Ljv, MG-Mena-9) using the Clustal Omega multiple sequence alignment tool (online version, October 2020), allowing to confirm the existence of molecules containing the expected deletions.

## 3. Results

### 3.1. Identification of Closteroviridae Members in Cassava

Thirteen contigs or scaffolds of more than 10 kilobases of *Closteroviridae* isolates could be reconstructed using the sequencing reads from the seven analyzed landraces (Table 4). The lengths of the contigs, which represent large parts of the corresponding genomes, ranged from 10,417 to 13,770 nucleotides (nt). Some of the analyzed plants contained multiple contigs corresponding to different isolates (Table 4). Thorough screening of these reconstructed contigs against viral reference databases revealed nucleotide and amino acid sequence homologies with members of the family *Closteroviridae* and, in particular, with the genus *Ampelovirus*. In addition to these previously unknown viruses, the results further indicated the presence of CBSVs and/or CMGs in three cassava samples (Table 1). Cassava brown streak virus (CBSV, isolate KOR6, GU563327) was detected in one of the plants from the DR Congo, while Uganda cassava brown streak virus (UCBSV, isolate KM) [20] and/or EACMV were detected in samples from Mayotte.

The isolates were named considering two letters of the code of the country where the landraces were collected, three letters of the landrace short acronym, and a number (when multiple contigs from the same landrace were obtained).

### 3.2. Genome Annotation

**Figure 1:**
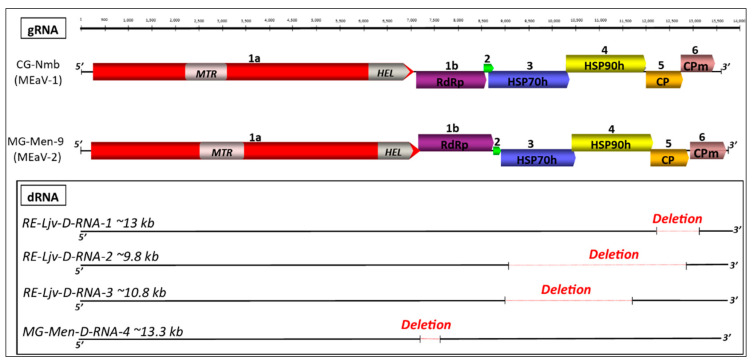
Schematic representation of the genomic organization of representative isolates CG-Nmb (MEaV-1) and MG-Men-9 (MEaV-2) (top) and structure of the defective variants (dRNA) identified (bottom). The RNA genome is drawn as a black line and the predicted open reading frames (ORFs) are represented by colored rectangles. Annotations and ORF numbers are given inside and above rectangles, respectively. Abbreviations: *MTR*—methyltransferase, *HEL*—helicase, *RdRp*—RNA-dependent RNA polymerase, *HSP70h*—heat shock protein 70 homolog, *HSP90h*— heat shock protein 90 homolog, *CP*—coat protein, *CPd*—minor coat protein.

The sequences comparisons between the various isolates identified two clearly separated groups (Figure 1). The nearly complete genome of a representative isolate was selected for each group (respectively, CG-Nmb for the MEaV-1 and MG-Men-9 for MEaV-2) and was analyzed in detail to determine the genome organization. As shown in Figure 1, this analysis predicted in both cases seven open reading frames encoding for polypeptides with a mass ranging from 6 to 258 kDa (Table 5). The genomic organization, the size, and the number of ORFs identified revealed a similarity with the subgroup II ampeloviruses. The recognized members of this subgroup are grapevine leafroll-associated virus 4 (GLRaV-4), pineapple mealybug wilt-associated virus 1 and 3 (PMWaV-1 and -3), air potato Ampelovirus 1 (AiPoV1), and plum bark necrosis and stem pitting-associated virus (PBNSPaV) [15].

The ORFs *1a* and *1b* encode the replication-associated proteins (Figure 1). As expected, a conserved domain search identified the following two replication-associated domains in the 1a protein: a methyltransferase motif (*MTR*; pfam01660) in the N-terminal part, and a viral helicase (*HEL*; superfamily 1, pfam01443) in the C-proximal part. The RNA methyltransferase domain was found in a wide range of ssRNA viruses and is known to be involved in mRNA capping.

The 1b ORF encodes the RNA-dependent RNA polymerase (*RdRp* 2 superfamily, pfam00978) expressed through a +1 ribosomal frameshift, as is seen in other members of the *Closteroviridae* family. Together with the *MTR* domain located at the 1a protein N-terminus, the *RdRp* forms the defining unique feature of the alpha-like ensemble of viruses [21].

Downstream of the polymerase, a small ORF2 encodes a hypothetical protein with a predicted molecular mass varying from 5 to 7 kDa depending on the isolate, and which is lacking known functional domains. The numbers and the sizes of the hypothetical proteins occurring downstream of the polymerase in the family *Closteroviridae* are highly variable. For example, Actinidia virus 1 (AcV-1) has three hypothetical proteins of predicted molecular masses of 13.6, 25.4 and 5.7 kDa [22]. However, in subgroup II ampeloviruses, only one hypothetical protein between 5 and 6 kDa is reported, sometimes overlapping with the 5′-proximal region of ORF3. The small size and the high proportion of the hydrophobic amino acids of the proteins encoded by the MG-Men-9 and CG-Nmb isolates (36/48 aa and 42/67 aa, respectively) could indicate that they are similar to the transmembrane proteins often present in the *Closteroviridae* members, where they have been proposed to function as cell-to-cell movement proteins [23].

ORF3 encodes a heat shock protein 70 homolog (*HSP70h*; cd10170) from the NBD–sugar–kinase–HSP70–actin superfamily. The functions postulated for *HSP70h* are as follows: the mediation of cell-to-cell movement through the plasmodesmata, involvement in the assembly of multisubunit complexes for genome replication and/or subgenomic RNAs synthesis, and the assembly of viral particles [9]. The size of this ORF is variable, being 62.2 kDa for MEaV-1 and 58.49 kDa for MEaV-2, as shown in the Supplementary Material (Table S1).

ORF4 codes for a heat shock protein 90 homolog (*HSP90h*, pfam03225), which is found to partially overlap with the 3′-proximal region of ORF3 and the 5′-proximal region of ORF5 as is the case for subgroup II ampeloviruses.

The coat protein (Closter coat superfamily, pfam01785) is encoded by ORF5.

It has been shown that downstream of the coat protein, some members of the family *Closteroviridae* have a variable number of ORFs encoding accessory proteins, some of which may have a functional conserved domain [24], while others show no or only very limited identity to any other proteins. The translated amino acid sequences of the protein encoded by the ORF6 have shown similarity with the p24 protein of the pineapple mealybug wilt-associated virus-1 (25.1%) and the minor coat protein (*CPd*) of the grapevine leafroll-associated virus-5 (20.8%) from the TrEMBL database of UniProt. This suggests that, although they share <20% identity with their respective, the proteins coded by the ORF6 could be considered as minor coat proteins. This ORF6 overlaps partially with the 3′-proximal region of ORF5.

All of the isolates sequenced share the same genome organization, although the contigs obtained for the isolates MG-Mia-403 and MG-Mian-2-2 were incomplete, missing ORF5 and ORF6.

The reads belonging to four defective RNA (D-RNAs) molecules were identified (Figure 1). Three of them were detected in the long Java accession (RE-Ljv) originating from the Reunion island (two for MEaV-1 (RE-Ljv-D-RNA-1 and RE-Ljv-D-RNA-2, accession numbers MW306827 and MW306828, respectively), one for MEaV-2 (RE-Ljv-D-RNA-3, accession number MW306829) and the remaining one in the Menatana accession originating from Madagascar (MG-Men-D-RNA-4 belonging to MEaV-2, accession number MW306830)). The deleted zones cover different genome regions and consist of most of the ORF5 and the ORF6 for the RE-Ljv-D-RNA-1, most of ORF3, ORF4, ORF5 and the start of ORF6 for the RE-Ljv-D-RNA-2, and most of the ORF3 and ORF4 for the RE-Ljv-D-RNA-3. In the case of the MG-Men-D-RNA-4, the deletion is at the beginning of the 1b ORF and does not alter the coding frame. The presence of molecules bearing these deletions in the original plant material was validated by the sequencing of amplicons spanning each of the individual deletions (see below). The proportion of deleted RNAs, as compared to the non-deleted genomic RNA, was estimated by comparing the read counts for the deletion borders and for the corresponding regions of the undeleted genome. This proportion was found to be variable, corresponding to about 4%, 37%, 27% and 3.8% for RE-Ljv-D-RNA-1, RE-Ljv-D-RNA-2, RE-Ljv-D-RNA-3 and MG-Men-D-RNA-4, respectively.

### 3.3. Phylogenetic Analysis of Cassava Isolates with Members of the Family Closteroviridae

A multiple alignment of the *HSP70h* protein of the thirteen isolates, and of the corresponding protein of known members of the family *Closteroviridae*, was used to perform pairwise amino acid comparisons and to generate a phylogenetic tree that allowed to first address the taxonomic position of the cassava isolates in the family *Closteroviridae*.

**Figure 2:**
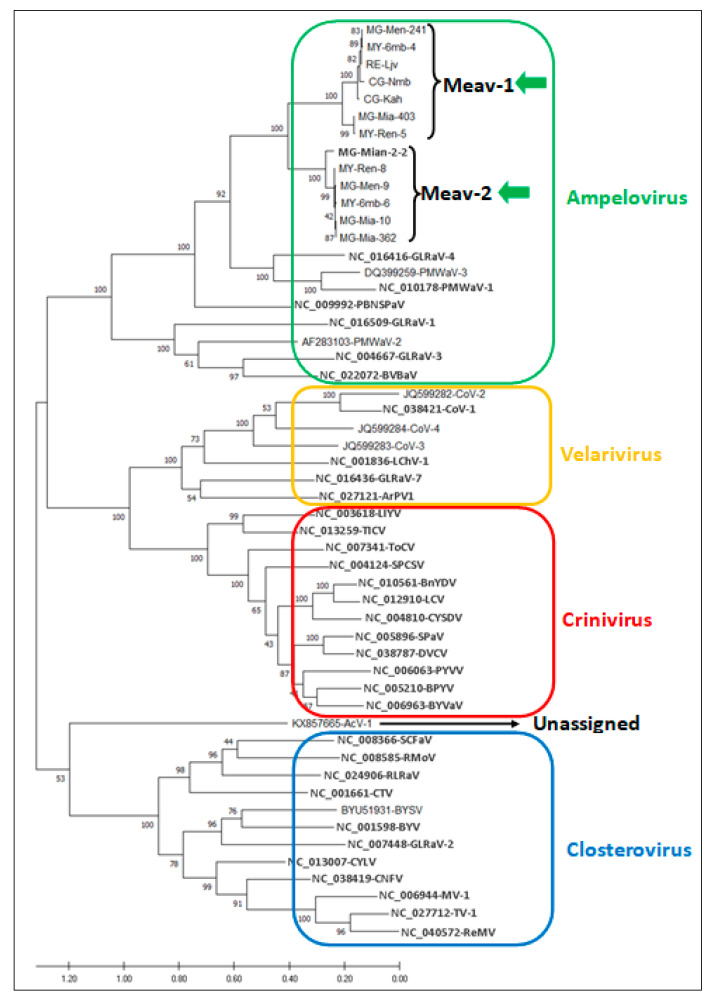
Phylogenetic analysis of the aligned amino acid sequences of the *HP70h* (ORF3) of the thirteen isolates from cassava and of selected members of the family *Closteroviridae* (see the Supplementary Material Table S2 for detailed information on these viruses). Green arrows indicate isolate sequences obtained in this study. Maximum likelihood phylogenetic tree was reconstructed in Mega X using Poisson model with uniform distribution for amino acid sequence alignments. Bootstrap values are indicated at the main branch nodes. The bar represents the number of amino acid substitution per site.

The resulting tree (Figure 2) placed them with strong bootstraps support in a clade with members of the genus *Ampelovirus*, specifically in the subgroup II clade adjacent to GLRaV-4 (NC_016416) and PMWaV-1 and 3 (NC_010178 and DQ_399259), confirming the taxonomical relatedness of these cassava isolates to the subgroup II of the *Ampelovirus* genus.

The molecular identity at the amino acid level for the *RdRp*, *HSP70h* and *CP* of the cassava isolates with members of the family *Closteroviridae* is systematically lower than 55% (Table 6), well below the 75% molecular species demarcation criterion for these taxonomically relevant genes in this family [9]. This shows that the isolates from cassava presented in this study are novel agents that do not belong to a known species in the family *Closteroviridae.*

### 3.4. Diversity of the New Isolates

**Figure 3:**
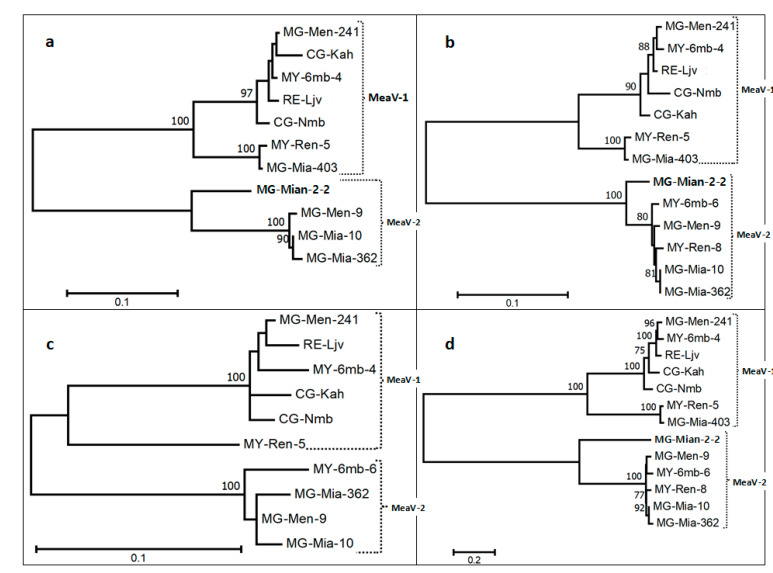
Phylogenetic trees reconstructed using the amino acid sequences of the following three taxonomically relevant proteins for the family *Closteroviridae*: (**a**) *RdRp*; (**b**) HSP70h; (**c**) *CP*; and (**d**) the whole genome nucleotide sequences. Maximum likelihood phylogenetic trees were reconstructed in Mega X using the GTR+GI model for nucleotide sequences alignments and a Poisson model with uniform distribution for amino acid sequence alignments. Only bootstrap values of more than 70% are mentioned on nodes. The scales provide branch distance for the given number of substitutions per site.

To investigate the phylogenetic relationships between the various reconstructed *Closteroviridae* genomic sequences from cassava, multiple alignments of the near-complete genomic sequences and of the amino acid sequences of the three taxonomically relevant proteins were used to reconstruct phylogenetic trees (Figure 3a–d).

From these analyses, it is clear that the cassava isolates form two well-separated phylogenetic groups (MEaV-1 and MEaV-2) that show 41.4 ± 0.3% average nucleotide divergence based on the whole genome and, respectively, 32.3 ± 1.7% (*RdRp*), 30.8 ± 2.0% (*HSP70h*) and 19 ± 2.2% (*CP*) average amino acid divergence (Table 6 and see the Supplementary Material Tables S3–S6 for additional information).

The first group (MEaV-1) clusters two isolates identified in two cassava landraces from Madagascar (MG-Men-241 and MG-Mia-403), one isolate identified in an accession from Reunion (RE-Ljv), two isolates identified in accessions from Mayotte (MY-6mb-4 and MY-Ren-5) and two isolates from DR Congo (CG-Nmb and CG-Kah). The following two isolates from this group are divergent from the others and form a distinct subgroup: MY-Ren-5 and MG-Mia-403. Indeed, the MEaV-1 average pairwise divergence is found to be 17.1 ± 0.2% based on the whole genome, and, respectively, 7.1 ± 0.7% (*RdRp*), 6.0 ± 0.6% (*HSP70h*) and 7.4 ± 1.0% (*CP*) for the various proteins (Table 6). However, if excluding the divergent isolates MG-Mia-403 and MY-Ren-5, these values fall down to 7.9 ± 0.2% for the whole genome and to 0.8 ± 0.4%(*RdRp*), 2.3 ± 0.4% (*HSP70h*) and 3.2 ± 0.6% (*CP*) for the various proteins, respectively. The average pairwise distance between these two divergent isolates, MG-Mia-403 and MY-Ren-5, is only 2.3% for the whole genome (Table 6).

The second group (MEaV-2) is composed of four isolates identified in two landraces from Madagascar (MG-Men-9, MG-Mia-2-2, MG-Mia-10 and MG-Mia-362) and two isolates identified in two landraces from Mayotte (MY-6mb-6 and MY-Ren-8). The isolate MG-Mian-2-2 forms a sub-cluster of its own. The MEaV-2 average pairwise divergence is 12.3 ± 0.1% for the whole genome, and, respectively, 7.3 ± 0.7% (*RdRp*) and 2.8 ± 0.4% (*HSP70h*) for the various genes (Table 7) (the average *CP* divergence was not computed because the *CP* gene is not covered by the MG-Mian-2-2 contig). If excluding the divergent MG-Mian-2-2 isolate, the average pairwise divergence for the whole genome falls to 3.8 ± 0.1%, and to 1.1 ± 0.3% (*RdRp*), 1.0 ± 0.3% (*HSP70h*) and 3.1 ± 0.8% (*CP*) for the various proteins, respectively (Table 7).

Comparisons across the trees based on the *RdRp, HSP70h* and *CP* proteins reveal variations in the branching topology that point to recombination events.

Considering the taxonomic differentiation between these two isolate groups (MEaV-1 and MEaV-2), largely supported by the phylogenetic trees, and the average divergence rates well over the 25% species demarcation criterion [14,15] for two of the three proteins (Table 6), it can be considered that the analyzed isolates form two distinct species, tentatively named “Manihot esculenta-associated ampelovirus 1 and 2” (MEaV-1 and MEaV-2), respectively.

**Figure 4:**
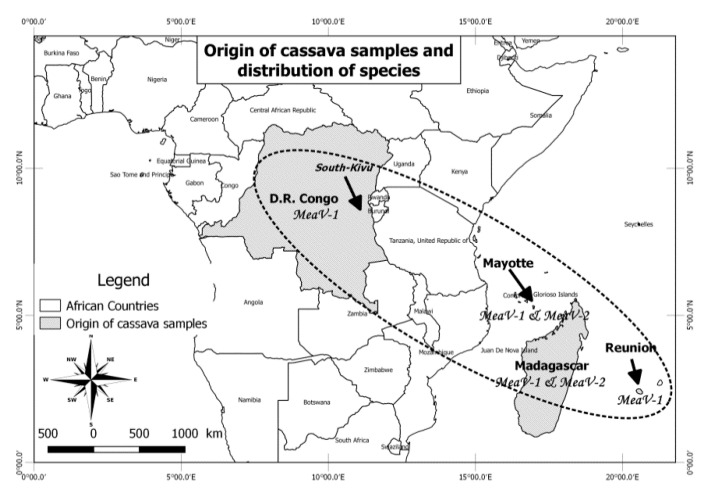
Geographic repartition of the two novel cassava ampelovirus species (MEaV-1 and MEaV-2) according to the country of origin of positive samples.

MEaV-1 is detected from the four regions included in the study, while MEaV-2 is observed only from Madagascar and Mayotte (Figure 4).

### 3.5. Recombination Analysis

Intraspecific and interspecific recombination events were investigated among the cassava isolates, and the recombination events supported that more than four of the nine algorithms integrated in RDP4 were considered as possible events [19].

**Figure 5:**
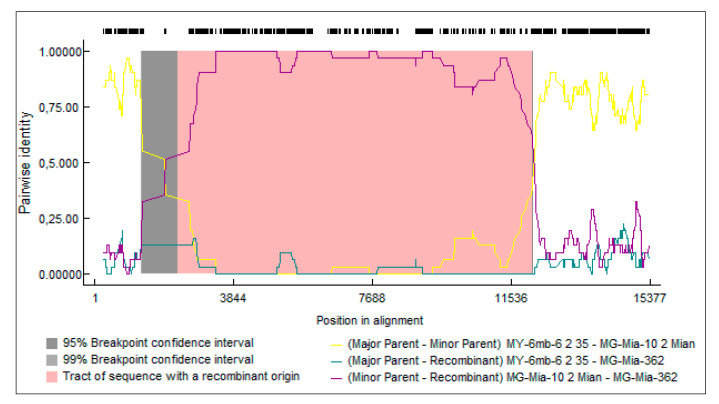
Recombination event detected among isolates from MeaV-2 by the RDP4 program.

Only one such recombination event was reliably detected using this criterion (Figure 5). This recombination event involves only the MEaV-2 isolates (two isolates MG-Mia-362 and MG-Mia-10 from Madagascar, and one isolate MY-6mb-6 from Mayotte) and is detected by seven out of the nine algorithms, with a strong probability (*p*-value = 2.75 × 10^−91^). The predicted recombination breakpoints are identified within ORF1a (nucleotide position 906 of the isolate MG-Mia-362) and within ORF4 (nucleotide position 8997 of the isolate MG-Mia-362).

### 3.6. Validation of Defective RNAs

**Figure 6:**
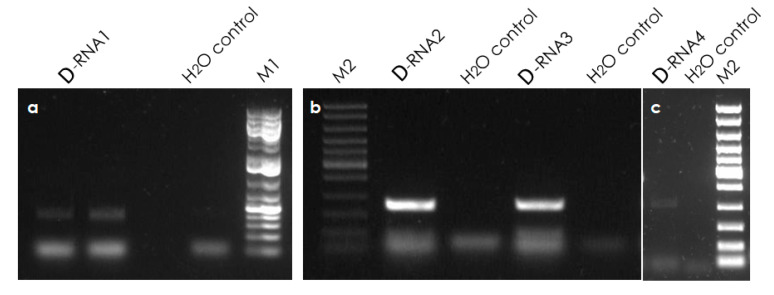
Gel red-stained 2% agarose gel showing RT-PCR products from: (**a**) D-RNA1 expected amplicon size 380 bp; (**b**) D-RNA2 and D-RNA3, expected amplicon size 380 bp and 400 bp, respectively; and (**c**) D-RNA4, expected amplicon size 500 bp. M1: GeneRuler 1 kb plus ladder (Thermo Scientific, SM1343). M2: fast DNA ladder 10 kb (NEB N3238S).

The presence of defective molecules was validated by the sequencing of RT-PCR amplicons spanning each of the identified deletions (Figure 6). The alignment of the four defective clones to their HTS-defective references, as shown in the Supplementary Material (Figures S7–S10), confirmed the presence of deletion zones at their predicted location. On the reference parental genomes (RE-Ljv and MG-Men-9), the deleted zones were found to be, respectively, 568 nt, 3761 nt, 648 nt and 444 nt for the D-RNA1, 2, 3 and 4.

These findings confirm that the D-RNAs reported here are not artifacts from high-throughput sequencing analysis, but continuous sequences that exist in the analyzed cassava samples.

## 4. Discussion

We have reported here the genome characterization from field-grown cassava plants from the DR Congo, Madagascar, Mayotte and Reunion of thirteen new *Closteroviridae* isolates, belonging to two potentially new ampelovirus species.

The analysis of the sequences of the corresponding contigs (ranging between 10,417 and 13,752 nucleotides in length) revealed seven open reading frames. In subgroup II, the length of the untranslated regions (UTRs) varies between 218 and 353 nt at the 5′ end, and between 125 and 132 nt at the 3′ end [25,26,27,28,29,30]. The UTR sizes reported here for the various genomes vary between 210 and 253 nt for the 5′end, and between 157 and 166 nt for the 3′ end. The comparison of these values, and the unambiguous assignation of MEaV-1 and MEaV-2 to the subgroup II of ampelovirus, show that the genomes reported here are unlikely to miss much more than a hundred nucleotides at their 5′ end and probably even less at the 3′ end, since some of the reported 3′ UTRs are already longer than the longest ones reported to date in the subgroup. This is confirmed when comparing the complete genome sizes; GLRaV-4 (isolate Man086, KJ810572) and PMWaV-1 (isolate HN, KJ872494) are the two subgroup II ampeloviruses with, respectively, the longest and the shortest genome (13,858 nt and 13,069 nt, respectively) [25,27]. The longest contigs for the representative isolates of MEaV-1 and MEaV-2 are, respectively, 13,616 nt (CG-Nmb) and 13,752 nt (MG-Men-9) long, suggesting that near-complete genomes, comprising the totality of the coding sequences and missing limited terminal non-coding nucleotide sequences, have been obtained, although the extremities of the genomes were not determined by RACE.

Based on the divergence of their *CPs*, which was determined to be below the species threshold (19 ± 2.2%), MEaV-1 and MEaV-2 could be considered as a single species. However, the official formulation of the taxonomic criterion for species demarcation in the family *Closteroviridae* and in ampelovirus genus is as follows: “Amino acid sequence of relevant gene products (polymerase, *CP* and *HSP70h*) differing by more than 25%” [14,15]. Therefore, there is an ambiguity on the fact of whether this criterion should be met for at least one of the three proteins or by all three simultaneously. Such a situation has previously been reported for Rehmannia virus 1 (ReV1) [31], for which the *RdRp* shows only 11% divergence with that of tobacco virus 1, while the *HSP70h* and the *CP* show 26% and 38% divergence, respectively. ReV1 was nevertheless accepted as a valid new *Closterovirus* species, suggesting that, as no recombination event was identified between them, MEaV-1 and MEaV-2 could similarly be considered as two distinct *Ampelovirus* species. This notion is further supported by the observation that all of the proteins diverge by more than 25%, between MEaV-1 and MEaV-2, except for the CP (and for a few comparisons involving one specific isolate, for the *HSP90h*).

Recombination is one of the mechanisms by which viruses evolve. Several studies have reported recombination events in *Closteroviridae* members [24,32,33]. In this study, we report a recombination event involving MEaV-2 isolates. No evidence of recombination between MEaV-1 and MEaV-2 was detected.

In most cases, the analyzed cassava plants contained complex mixed infections involving either several isolates from a single MEaV species, or isolates belonging to the two species. Such complex infection patterns are probably due to the vegetative propagation practices used for cassava cultivation. In two plants with these complex mixed infections, defective molecules were identified and confirmed by RT-PCR, cloning and sequencing. Defective RNAs belong to the category of virus-associated molecules that are not required for normal virus propagation, but can sometimes affect the accumulation of the helper virus and symptoms expression [34,35,36]. The presence of defective RNAs is reported in three genera of the *Closteroviridae* family [28,37,38,39,40], and is therefore not unexpected here. The considerable advances in virus characterization by HTS-based approaches are now revealing that, in addition to genomic and subgenomic RNAs, plants infected with viruses from the family *Closteroviridae* may contain several different subviral defective RNAs whose role is not known [35]. Further studies are needed to assess their impact on epidemiology and pathogenicity.

The sampling campaign conducted in the DR Congo collected stems/cuttings from cassava plants showing typical symptoms of CBSD on their leaves and stems. These symptoms consisted of a yellow blotchy pattern on mature lower leaves and brown–black marks (‘streaks’) on green stem portions. The collected cuttings were planted in a glasshouse and, after approximately six months, two of the planted cuttings that have previously expressed foliar symptoms in a farmer’s field remained asymptomatic in the glasshouse and were used for the present study. The HTS results have confirmed the presence of CBSV in one of the samples, but not in the second one. Furthermore, the typical symptoms of CBSD and/or CMD that were observed on the samples from Mayotte are to be connected, in both cases, to the identification of the corresponding causal virus(es). No virus-like symptoms were observed on the samples from Reunion or from Madagascar, the Reunion sample being even selected in this study as a healthy control plant. Additional experiments of artificial inoculation and larger epidemiological studies are needed to clarify the symptomatology of MEaV-1 and MEaV-2, and to estimate the synergistic interaction between coinfecting viruses such as CBSVs and/or CMGs. The symptoms mentioned in this study consist only of foliar symptoms, while no observation was made on below-ground organs.

There is a need to develop a diagnostic test in order to be able to evaluate the distribution and prevalence of these new viral agents in other regions of the world, and to evaluate their impact on the yield. This evaluation of the phytosanitary risk should further be completed through wider surveys on symptomatic and asymptomatic plants in both agricultural and natural ecosystems to gain insight into the genetic variability of these new viruses, and their biological significance and impact [41].

## Figures and Tables

**Table 1:** Identity and origin of cassava landraces used and indication on the high-throughput sequencing (HTS) protocol performed on these samples.

Cassava Landraces	Country of Origin	Collection Date DD/MM/YY	Symptoms	Sequencing Strategy and Target	Isolates Detected	Other Virus Detected
Menatana	Madagascar	18/10/2017	Asymptomatic in greenhouse	VANA	MG-Men-241, MG-Men-9	-
Miandrazaka	Madagascar	18/10/2017	Asymptomatic in greenhouse	VANA	MG-Mia-10, MG-Mia-362, MG-Mia-403, MG-Mian 2-2	-
Long Java	Reunion	25/02/2015	Asymptomatic in field	dsRNAs	RE-Ljv	-
Reunion	Mayotte	26/03/2015	Symptoms of CMD and CBSD	dsRNAs	MY-Ren-5, MY-Ren-8	EACMV+UCBSV
6 Mois Blanc	Mayotte	26/03/2015	Symptoms of CMD	dsRNAs	MY−6mb-4, MY−6mb-6	EACMV
Nambiyombiyo	DR Congo	13/01/2016	Asymptomatic in greenhouse	Total RNA	CG-Nmb	CBSV
Kahunde	DR Congo	13/01/2016	Asymptomatic in greenhouse	Total RNA	CG-Kah	-

VANA: virion-associated nucleic acid; dsRNAs: double-stranded RNAs; EACMV: East African cassava mosaic virus; UCBSV: Ugandan cassava brown streak virus; CBSV: cassava brown streak virus; CMD: cassava mosaic disease; CBSD: cassava brown streak disease.

**Table 2:** RT-PCR primer pairs used for the confirmation of the viral sequences in the samples.

PRIMER	TARGET ORF	SEQUENCES (5′==> 3′)	Tm
12.495 F cp	CP ORF5	AATTTGGGAGGAGTGCGACC	60.3
12.715 R cp	CP ORF5	AGACCGACTTGTGCTACTCTTG	60.0
11.735 F hsp90	HSP90 ORF4	GTCCCGGCCTACATGCAATT	60.8
11.959 R hsp90	HSP90 ORF4	ACCGCCAGTCAACTCTCGTA	60.9
9.021 F hsp70	HSP70 ORF4	TATGGTTGTTGCTCGCGACT	60.0
9.292 R hsp70	HSP70 ORF4	CTGACAAACCAGCAGCAGTTG	60.3
7.244 F rdrp	RdRp ORF1B	GGACAACCTCCGAAACCGTAT	60.1
7.544 R rdrp	RdRp ORF1B	CTTTCGCTGCCATTGGTGTC	60.1

**Table 3:** RT-PCR primer pairs used for the characterization of the defective RNA molecules.

Primers Name	Sequences (5’==>3’)	Expected Amplicon Size
MEaV-1-RE-Ljv-D-RNA1-F	ACATCTAAATGCTAACGAACGAAGAG	380 bp
MEaV-1-RE-Ljv-D-RNA1-R	CAACGCCAGAATCTTCGTACA	
MEaV-1-RE- Ljv-D-RNA2-F	AGGCTTTCGACAGTGAAGAAGTG	330 bp
MEaV-1-RE- Ljv-D-RNA2-R	CGTAGCCATACTGAAGGATAGCA	
MEaV-2-RE- Ljv-D-RNA3-F	TGGAAGCCGCTGGTAAACTACA	400 bp
MEaV-2-RE-Ljv-D-RNA3-R	CAAGCACGTTCAATATTAGGAATAGTAC	
MEaV-2-MG-Mena-D-RNA4-F	AGACATATGAAAGAGTTGCATTGGTG	500 bp
MEaV-2-MG-Mena-D-RNA4-R	ACCTACAAATAATTTCGCTCGTCTG	

**Table 4:** Lengths, number of reads integrated, percent of total reads and average coverage for *Closteroviridae* contigs from the various analyzed cassava samples.

Cassava Landraces	Isolate Name ^1^	Accession Number (Genbank)	Virus Name	Contig Length [Nt]	Number of Reads Mapped	Percent Of Total Reads	Average Genome Coverage	Internal Undetermined Nucleotides	Internal Gaps
Menatana	MG-Men-241	MT773584	MEaV-1	13,528	38,475	12.1	506.8X	0	0
	MG-Men-9	MT773591	MEaV-2	13,752	10,945	4.10	153.6X	0	0
Miandrazaka	MG-Mia-10	MT773592	MEaV-2	13,770	26,987	4.8	368X	377	1
	MG-Mia-362	MT773594	MEaV-2	12,007	19,421	8.24	322X	0	0
	MG-Mia-403	MT773590	MEaV-1	10,518	26,917	11.4	480.9X	0	0
	MG-Mian 2-2	MT773596	MEaV-2	10,417	7676	1.4	136.4X	843	1
Long java	RE-Ljv	MT773586	MEaV-1	13,640	21,294	18.5	312.5X	0	0
Reunion	MY-Ren-5	MT773589	MEaV-1	13,172	1976	3.4	29.6X	72	1
	MY-Ren-8	MT773595	MEaV-2	13,762	377	0.7	5.4X	2889	14
6 Mois blanc	MY-6mb-4	MT773585	MEaV-1	13,615	4992	12.7	73.3X	288	4
	MY-6mb-6	MT773593	MEaV-2	13,764	2631	6.7	39.2X	990	9
Nambiyombiyo	CG-Nmb	MT773587	MEaV-1	13,612	7502	0.12	42X	0	0
Kahunde	CG-Kah	MT773588	MEaV-1	13,616	15,351	0.23	75X	11	1

**^1^** Isolates were named considering two letters of the code of the country where the landraces were collected—three letters of the landrace short acronym—and a number (when multiple contigs from the same landrace were obtained)

**Table 5:** Genome length, position of ORFs and size of the encoded proteins for the representative isolates of the two species CG-Nmb (MEaV-1) and MG-Men-9 (MEaV-2).

Isolates	Genome Length (Nt)	Length of Open Reading Frames (Orfs) (Nt) and Molecular Mass of Encoded Proteins (Kda)						
		*1a*	*1b*	*P5-P7*	*HSP70h*	*HSP90h*	*CP*	ORF6
**CG-Nmb** (MEaV-1)	13,616 nt	6789 nt	1503 nt	204 nt	1707 nt	1686 nt	774 nt	717 nt
		254.6 kDa	56.6 kDa	7.6 kDa	62.2 kDa	63.4 kDa	27.7 kDa	27.1 kDa
**MG-Men-9** (MEaV-2)	13,752 nt	6975 nt	1593 nt	147 nt	1608 nt	1698 nt	816 nt	639 nt
		258.8 kDa	60.4 kDa	5.3 kDa	58.4 kDa	64.3 kDa	29.3 kDa	24 kDa

**Table 6:** Amino acid sequence identity of the RdRp, HSP70h and CP of MEaV-2 (isolate MG-Men-9) with those of other selected *Closteroviridae* members.

Genus	Representative Members	Proteins		
		*RdRp*	*HSP70H*	*CP*
***Ampelovirus***	MEaV-1 (isolate CG-Nmb)	66.5%	69.9%	80.1%
***Ampelovirus***	Grapevine leafroll-associated virus 4 (NC_016416)	41.7%	51.6%	47.6%
***Closterovirus***	Citrus tristeza virus (NC_001661)	27.2%	25%	12.5%
***Crinivirus***	Potato yellow vein virus (NC_006062 and NC_006063)	25.7%	26.5%	17.2%
***Velarivirus***	Grapevine leafroll-associated virus 7 (NC_016436)	27.5%	27.7%	13.6%

**Table 7:** Intergroup and intragroup average pairwise divergence and standard deviation calculated for the three taxonomically relevant proteins (*RdRP, HSP70h and CP*) and for the nearly complete genomes.

		Within MEaV-2 with Mian 2-2	Within MEaV-2 without Mian2-2	Within MEaV-1 with Divergents	Within MEaV-1 without Divergents	Within MEaV-1 Divergents	Between MEaV-1 and MEaV-2
*RdRp*	aa divergences	7.3 +/− 0.7%	1.1 +/− 0.3%	7.1 +/− 0.7%	0.8 +/− 0.4%	0.8%	32.3 +/− 1.7%
*HSP70h*	aa divergences	2.8 +/− 0.4%	1.0 +/− 0.3%	6.0 +/− 0.6%	2.3 +/− 0.4%	0.7%	30.8 +/− 2.0%
*CP*	aa divergences	na	3.1 +/− 0.8%	7.4 +/− 1.0%	3.2 +/− 0.6%	na	19 +/− 2.2%
Genome	nt divergences	12.3 +/− 0.1%	3.8 +/− 0.1%	17.1 +/− 0.2%	7.9 +/− 0.2%	2.3%	41.4 +/− 0.3%

MEaV-1 divergents: MY-Ren-5 and MG-Mia-403. na: information not available as the contig/scaffold does not extend to the region concerned.

## Data Availability

The datasets of genome sequences generated and analyzed during this study are available in Genbank repository under the following accession numbers: MT773584, MT773585, MT773586, MT773587, MT773588, MT773589, MT773590, MT773591, MT773592, MT773593, MT773594, MT773595, MT773596, MW306827, MW306828, MW306829, MW306830.

## Supplementary Materials

The following are available online at https://www.mdpi.com/article/10.3390/v13061030/s1; Figure S1: multiple sequence alignment of D-RNA1 clone (referred to in the figure as D-RNA1_Sanger) with the defective reference from HTS (MEaV-1_RE-LJV-D-RNA-1). The term “DELETIONZONE” has been inserted into the HTS-defective reference to identify the zone where the deletion is located. This zone consists of 568 nucleotide sequences and is indicated by the rectangle. Figure S2: multiple sequence alignment of D-RNA2 clone (referred to in the figure as D-RNA2_Sanger) with the defective reference from HTS (MEaV-1_RE-LJV-D-RNA-2). The term “DELETIONZONE” has been inserted into the HTS-defective reference to identify the zone where the deletion is located. This zone consists of 3761 nucleotide sequences and is indicated by the rectangle. Figure S3: multiple sequence alignment of D-RNA3 clones (two clones were sequenced and are referred to in the figure as D-RNA3a-Sanger and D-RNA3b-Sanger) with the defective reference from HTS (MEaV-2_RE-LJV-D-RNA3). The term “DELETIONZONE” has been inserted into the reference HTS-defective to identify the zone where the deletion has been identified. This zone consists of 648 nucleotide sequences and is indicated by the rectangle. Figure S4: multiple sequence alignment of D-RNA4 clone (referred to in the figure as D-RNA4-Sanger) with the defective reference from HTS (MEaV-2_MG-Mena-D-RNA-4). The term “DELETIONZONE” has been inserted into the reference HTS-defective to identify the zone where the deletion is located. This zone consists of 444 nucleotide sequences and is indicated by the rectangle. Table S1: length of ORF and molecular mass of 3 encoded proteins (RdRp, HSP70h and CP) of the 15 isolates of the cassava ampelovirus reported in this study. Table S2: accession numbers and abbreviations for selected Closterovirus used in the phylogenetic reconstruction of the HSP70h nucleotide sequences. Table S3: estimates of evolutionary divergence between amino acid (aa) sequences for the HSP70h protein among cassava ampeloviruses isolates reported in this study. Table S4: estimates of evolutionary divergence between amino acid (aa) sequences for the HSP70h protein among cassava ampeloviruses isolates reported in this study. Table S5: estimates of evolutionary divergence between amino acid (aa) sequences for the coat protein among cassava ampeloviruses isolates reported in this study. Table S6: estimates of evolutionary divergence between nucleotide sequences for the whole genomes of cassava ampelovirus isolates reported in this study.
