# Supplementary material for: Novel Ampeloviruses Infecting Cassava in Central Africa and the South-West Indian Ocean Islands
Source: Viruses. 2021 May 29;13(6):1030. doi: 10.3390/v13061030 (PMC8226816; doi:10.3390/v13061030)
Supplement: Supplementary file 1 [file viruses-13-01030-s001.zip › Figure S1 & Figure S2 & Figure S3 & Figure S4.pdf]

```
Meav-1_RE-LJV-D-RNA1      ACATGCAATTCGATTTTGCGGTGGCCTTGGATCCTCTACATCTAAATGCTAACGAACGAA  11813
D-RNA1_Sanger             --------------------------------------------AATGCTAACGAACGAA  61
                                                                      ****************

Meav-1_RE-LJV-D-RNA1      GAGCTTTGACTTCATTGTTAGCTAGATTTAGAACCAGGAGAACACCTGTCAGAGGGCAAG  11873
D-RNA1_Sanger             GAGCTTTGACTTCATTGTTAGCTAGATTTAGAACCAGGAGAACACCTGTCAGAGGGCAAG  121
                          ************************************************************

Meav-1_RE-LJV-D-RNA1      CCTTAGGACAACGTGCTAGCAATCCACTAGATGATGTGGTTGAATCTATAGTTCCAGCGA  11933
D-RNA1_Sanger             CCTTAGGACAACGTGCTAGCAATCCACTAGATGATGTGGTTGAATCTATAGTTCCAGCGA  181
                          ************************************************************

Meav-1_RE-LJV-D-RNA1      CTTCTCGAGGAATGTTACGAGAGTTGACTGGCGGTGCCAGTACGCTTGGGTATAATAGAA  11993
D-RNA1_Sanger             CTTCTCGAGGAATGTTACGAGAGTTGACTGGCGGTGCCAGTACGCTTGGGTATAATAGAA  241
                          ************************************************************

Meav-1_RE-LJV-D-RNA1      GAAGACGCTACTAATTCAGCACCATGGCAACACCAACACCTGGAACACCAAACTCTACTC  12053
D-RNA1_Sanger             GAAGACGCTACTAATTCAGCACCATGGCAACACCAACACCTGGAACACCAAACTCTACTC  301
                          ************************************************************

Meav-1_RE-LJV-D-RNA1      CTCCTGATGCAAACAGTACTAATACCAACAGTCAAGTTGTGGCTGCTGTCGTACCTGGAG  12113
D-RNA1_Sanger             CTCCTGATGCAAACAGTACTAATACCAACAGTCAAGTTGTGGCTGCTGTCGTACCTGGAG  361
                          ************************************************************

Meav-1_RE-LJV-D-RNA1      GTCGTCAGACCGTTGDELETIONZONEAATAAACAAGAGTAGCACAAGTCGGTCCAACCC  12173
D-RNA1_Sanger             GTCGTCAGACCGTTG------------AATAAACAAGAGTAGCACAAGTCGGTCCAACCC  409
                          ***************            *********************************

Meav-1_RE-LJV-D-RNA1      TCACAATATGTACGAAGATTCTGGCGTTGCTCCAGAAATTTTCATGGGAGGACGACGTTG  12229
D-RNA1_Sanger             TCACAATATGTACGAAGATTCTGGCGTTG-------------------------------  465
                          *****************************
```

Figure S1: Multiple sequence alignment of D-RNA1 clone (referred to in the figure as D-RNA1_Sanger) with the defective reference from HTS (MEaV-1_RE-LJV-D-RNA-1). The term "DELETIONZONE" has been inserted into the HTS defective reference to identify the zone where the deletion is located. This zone consists in 568 nucleotide sequences and is indicated by the rectangle.

```
Meav-1_RE-LJV-D-RNA2      GAAGGAGCGTGCAGTAAAACTCTAATGCGTGAAAAATGGGTGTCTTTCAATGACATTACT  8400
D-RNA2_Sanger             ------------------------------------------------------------  7


Meav-1_RE-LJV-D-RNA2      CAGGCTTTCGACAGTGAAGAAGTGTGTTGTCGTCTAGCCGCGTTGTGTGAGGAGAAATAC  8460
D-RNA2_Sanger             ------TTCGACAGTGAAGAAGTGTGTTGTCGTCTAGCCGCGTTGTGTGAGGAGAAATAC  67
                                ******************************************************

Meav-1_RE-LJV-D-RNA2      GGTCCTAGTCCGTGGTGGTACGCTGCGATAGCGACAATTCATTGTATCAGGGCCAATCCG  8520
D-RNA2_Sanger             GGTCCTAGTCCGTGGTGGTACGCTGCGATAGCGACAATTCATTGTATCAGGGCCAATCCG  127
                          ************************************************************

Meav-1_RE-LJV-D-RNA2      CAGCAATTTGAGAGGTGTTGGAGTGTAATATCTTCTCCTAGTATAAATGAAGCAAAGAAG  8580
D-RNA2_Sanger             CAGCAATTTGAGAGGTGTTGGAGTGTAATATCTTCTCCTAGTATAAATGAAGCAAAGAAG  187
                          ************************************************************

Meav-1_RE-LJV-D-RNA2      AGAAGCAAGGTCGTCTCCGAAGGAGGACCTGCGAGTTGTACAAATGACCAATTTATATTT  8640
D-RNA2_Sanger             AGAAGCAAGGTCGTCTCCGAAGGAGGACCTGCGAGTTGTACAAATGACCAATTTATATTT  247
                          ************************************************************

Meav-1_RE-LJV-D-RNA2      TGATTTTAAGTGGGCTATAACCGTTTDELETIONZONEACCTACAATTATGATGAAATAA  8700
D-RNA2_Sanger             TGATTTTAAGTGGGCTATAACCGTTT------------ACCTACAATTATGATGAAATAA  295
                          **************************            **********************

Meav-1_RE-LJV-D-RNA2      AAACGACCATTGATAATGCTATCCTTCAGTATGGCTACGAAAACCCCGTTCGCCAATTCG  8760
D-RNA2_Sanger             AAACGACCATTGATAATGCTATCCTTCAGTATGGCTAC----------------------  337
                          **************************************

Meav-1_RE-LJV-D-RNA2      GGAGGAGTGCGACCAGTTTCATAATTCAAGCATTAGCAAATGGACTCATGGAACCCAATG  8820
D-RNA2_Sanger             ------------------------------------------------------------  337
```

Figure S2: Multiple sequence alignment of D-RNA2 clone (referred to in the figure as D-RNA2_Sanger) with the defective reference from HTS (MEaV-1_RE-LJV-D-RNA-2). The term “DELETIONZONE” has been inserted into the HTS defective reference to identify the zone where the deletion is located. This zone consists in 3761 nucleotide sequences and is indicated by the rectangle.

```
Meav-2_RE-LJV-D-RNA3      CATTCCACTCCGTACTTCTGTTCGAAGTTCTTGGTGGAAGCCGCTGGTAAACTACATTAC  8460
D_RNA3a-Sanger            --------------------------------------------------ACTACATTAC  10
D_RNA3b-Sanger            --------------------------------------------------ACTACATTAC  10
                                                                            **********

Meav-2_RE-LJV-D-RNA3      GTACCCGACCCCTTCAAAATTTTTATAAAATTTGGAGCGGAAGGTGCACCGACAAAGCCA  8520
D_RNA3a-Sanger            GTACCCGACCCCTTCAAAATTTTTATAAAATTTGGAGCGGAAGGTGCACCGACAAAGCCA  70
D_RNA3b-Sanger            GTACCCGACCCCTTCAAAATTTTTATAAAATTTGGAGCGGAAGGTGCACCGACAAAGCCA  70
                          ************************************************************

Meav-2_RE-LJV-D-RNA3      TTATTAAAAGAGAAGTGGGCTTCATTTTACGACATAACAACTGCTTATGACAGTGAGGAA  8580
D_RNA3a-Sanger            TTATTAAAAGAGAAGTGGGCTTCATTTTACGACATAACAACTGCTTATGACAGTGAGGAA  130
D_RNA3b-Sanger            TTATTAAAAGAGAAGTGGGCTTCATTTTACGACATAACAACTGCTTATGACAGTGAGGAA  130
                          ************************************************************

Meav-2_RE-LJV-D-RNA3      GTCTGCATCAAGTTGGCCCATCTATGCGCTGAGAAATACGGTGCATCGAATTGGTGGTAC  8640
D_RNA3a-Sanger            GTCTGCATCAAGTTGGCCCATCTATGCGCTGAGAAATACGGTGCATCGAATTGGTGGTAC  190
D_RNA3b-Sanger            GTCTGCATCAAGTTGGCCCATCTATGCGCTGAGAAATACGGTGCATCGAATTGGTGGTAC  190
                          ************************************************************

Meav-2_RE-LJV-D-RNA3      GCTGCTATAGCTACAATCCATTGTATTCGTGCAAACCCAACACAATTTGCACGATGTTGG  8700
D_RNA3a-Sanger            GCTGCTATAGCTACAATCCATTGTATTCGTGCAAACCCAACACAATTTGCACGATGTTGG  250
D_RNA3b-Sanger            GCTGCTATAGCTACAATCCATTGTATTCGTGCAAACCCAACACAATTTGCACGATGTTGG  250
                          ************************************************************

Meav-2_RE-LJV-D-RNA3      GAGACAGTTGATTGTGAGAGCATACGTTCTCGTAAGTCGDELETIONZONECAAACAAAC  8760
D_RNA3a-Sanger            GAGACAGTTGATTGTGAGAGCATACGTTCTCGTAAGTCG------------CAAACAAAC  298
D_RNA3b-Sanger            GAGACAGTTGATTGTGAGAGCATACGTTCTCGTAAGTCG------------CAAACAAAC  298
                          ***************************************            *********

Meav-2_RE-LJV-D-RNA3      TAGTGTGGCAGTGGGGTGACAAAACCGTTACTACTACTTATAATAGTATTTCCGCTATGT  8820
D_RNA3a-Sanger            TAGTGTGGCAGTGGGGTGACAAAACCGTTACTACTACTTATAATAGTATTTCCGCTATGT  358
D_RNA3b-Sanger            TAGTGTGGCAGTGGGGTGACAAAACCGTTACTACTACTTATAATAGTATTTCCGCTATGT  358
                          ************************************************************

Meav-2_RE-LJV-D-RNA3      TTGCGAAACATGGAGGTACTATTCCTAATATTGAACGTGCTTGGTGCAATCCACTAGCTT  8880
D_RNA3a-Sanger            TTGCGAAACATGGAGGTACTATTCCTAATATTGAACGTGCTTGATCTT------------  406
D_RNA3b-Sanger            TTGCGAAACATGGAGGTACTATTCCTAATATTGAACGTGCTTGATCTT------------  406
                          ****************************************** *
```

Figure S3: Multiple sequence alignment of D-RNA3 clones (two clones were sequenced and are referred to in the figure as D-RNA3a-Sanger and D-RNA3b-Sanger) with the defective reference from HTS (MEaV-2_RE-LJV-D-RNA3). The term "DELETIONZONE" has been inserted into the reference HTS defective to identify the zone where the deletion has been identified. This zone consists in 648 nucleotide sequences and is indicated by the rectangle.

```
Meav-2_MG-Mena-D-RNA-4      CGAGTGGCCACTGTGCATGAGTGTCAAGGGAAGACATATGAAAGAGTTGCATTGGTGCGA      5100
D-RNA4_Sanger               ------------------------------------------------------------      0

Meav-2_MG-Mena-D-RNA-4      ATCAAGCCCGCACAAGACGAAGTCTTCACGAGCGCTCCACATAGATTAGTGGCTCTCACT      5160
D-RNA4_Sanger               -------AGCCGCAAGACGAAGTCTTCACGAGCGCTCCACATAGATTAGTGGCTCTCACT      53
                                      * ************************************************

Meav-2_MG-Mena-D-RNA-4      CGACACACTACGAGTTTAGACTTCTATTGCATAAGAAATCGAATGGACCAGGGGATTGGT      5220
D-RNA4_Sanger               CGACACACTACGAGTTTAGACTTCTATTGCATAAGAAATCGAATGGACCAGGGGATTGGT      113
                            ************************************************************

Meav-2_MG-Mena-D-RNA-4      CGAGATGTCGAACTGATTGAGAAAATAACGGAACACGTCGCGAGGACTTTCTTAATTGAG      5280
D-RNA4_Sanger               CGAGATGTCGAACTGATTGAGAAAATAACGGAACACGTCGCGAGGACTTTCTTAATTGAG      173
                            ************************************************************

Meav-2_MG-Mena-D-RNA-4      CAGTGTTCCTAACAATTATTACGTDELETIONZONETGATTTAGCAGAATGGACCATGTC      5340
D-RNA4_Sanger               CAGTGTTCCTAACAATTATTACGT------------TGATTTAGCAGAATGGACCATGTC      221
                            ************************            ************************

Meav-2_MG-Mena-D-RNA-4      CAGAACGACATCGGGTAAGGCAGGTTTGTTACAAAAACTGCAAATACCTTCACTTCCCCA      5400
D-RNA4_Sanger               CAGAACGACATCGGGTAAGGCAGGTTTGTTACAAAAACTGCAAATACCTTCACTTCCCCA      281
                            ************************************************************

Meav-2_MG-Mena-D-RNA-4      TACTCAACTGGATGAATTCAAACTTATGGTGAAAGGGGATTTGAAACCAAAACTAGATGA      5460
D-RNA4_Sanger               TACTCAACTGGATGAATTCAAACTTATGGTGAAAGGGGATTTGAAACCAAAACTAGATGA      341
                            ************************************************************

Meav-2_MG-Mena-D-RNA-4      CACTTGCTTATCCGACATTCCGAGTGGTCAAAATATAGTTTATCACGAACGAGCAGTTTG      5520
D-RNA4_Sanger               CACTTGCTTATCCGACATTCCGAGTGGTCAAAATATAGTTTATCACGAACGAGCAGTTTG      401
                            ************************************************************

Meav-2_MG-Mena-D-RNA-4      TGCTTGTTTTTCTGTCTGTTTCATCCAAATGACAAAAAGATTGAAAGCCATATTAAACAG      5580
D-RNA4_Sanger               TGCTTGTTTTTCTGTCTGTTTCATCCAAATGACAAAAAGATTGAAAGCCATATTAAACAG      461
                            ************************************************************

Meav-2_MG-Mena-D-RNA-4      ACGAGCGAAATTATTTGTAGGTGTAGACCTCTCTGAATTTGCTCTCGATATTCAGCTCAC      5640
D-RNA4_Sanger               ACGAGCGAAATTATTTGTAGGTAA------------------------------------      485
                            **********************
```

Figure S4: Multiple sequence alignment of D-RNA4 clone (referred to in the figure as D-RNA4-Sanger) with the defective reference from HTS (MEaV-2_MG-Mena-D-RNA-4). The term “DELETIONZONE” has been inserted into the reference HTS defective to identify the zone where the deletion is located. This zone consists in 444 nucleotide sequences and is indicated by the rectangle.
