# Supplementary material for: Novel Ampeloviruses Infecting Cassava in Central Africa and the South-West Indian Ocean Islands
Source: Viruses. 2021 May 29;13(6):1030. doi: 10.3390/v13061030 (PMC8226816; doi:10.3390/v13061030)
Supplement: Supplementary file 1 [file viruses-13-01030-s001.zip › Table S1.pdf]

Table S1: Length of ORF and molecular mass of 3 encoded proteins (RdRp, HSP70h and CP) of the 15 isolates of the cassava ampelovirus reported in this study.

Species	Isolates	Genome Length (nt)	Length of ORFs for predicted proteins (nt)/Molecular Mass of encoded proteins (kDa)		
			RdRp	HSP70h	CP
MeaV-1	MG-Men-241	13,528	1,521/57.4	1,707/62.1	774/27.8
	RE-LJV	13,640	1,521/57.3	1,707/62.7	774/27.7
	MG-Mia-403	**na**	1,653/60	1,671/58.6	**na**
	MY-6mb-4	13,615	1,500/56.5	1,707/60.6	774/26.9
	CG-Nmb	13,612	1,503/56.6	1,707/62.2	774/27.7
	CG-Kah	13,616	1,521/57.4	1,707/61.7	774/27.7
	MY-Reu-5	13,636	1,614[a]/57.8	1,707/62.3	768/27.6
MeaV-2	MG-Mian-2-2	**na**	16,53/60.3	16,71/58.2	**na**
	MG-Men-9	13,752	15,93/60.4	16,08/58.5	816/27.9
	MG-Mia-10	13,770	15,93/60.3	16,08/58.5	816/27.9
	MY-6mb-6	13,764	15,54/42.3	16,08/58.4	816/27.9
	MY-Reu-8	**na**	15,93/49.3	16,08/55.8	**na**
	MG-Mia-362	12,007	15,93/60.3	16,08/58.4	768/27.7

na: information not available as the contig/scaffold does not extend to the region concerned.

[a]: The ORF is whether truncated or has gaps.
