# Supplementary material for: Novel Ampeloviruses Infecting Cassava in Central Africa and the South-West Indian Ocean Islands
Source: Viruses. 2021 May 29;13(6):1030. doi: 10.3390/v13061030 (PMC8226816; doi:10.3390/v13061030)
Supplement: Supplementary file 1 [file viruses-13-01030-s001.zip › Table S2.pdf]

Table S2: Accession numbers and abbreviations for selected closterovirus used in the phylogenetic reconstruction of the HSP70h nucleotide sequences.

Genus	Species Names	Assigned abbreviation	Sequence accession N° (GenBank)
Closterovirus	beet yellow stunt virus	(BYSV)	[BYU51931]
	beet yellow virus	(BYV)	[NC_001598]
	Citrus tristeza Virus	(CTV)	[NC_001661]
	mint virus 1	(MV-1)	[NC_006944]
	grapevine leafroll-associated virus 2	(GLRaV-2)	[NC_007448]
	strawberry chlorotic fleck associated virus	(SCFaV)	[NC_008366]
	raspberry mottle virus	(RMV)	[NC_008585]
	carrot yellow leaf virus	(CYLV)	[NC_013007]
	rose leaf rosette-associated virus	(RLRaV)	[NC_024906]
	tobacco virus 1	(ToV-1)	[NC_027712]
	carnation necrotic fleck virus	(CNFV)	[NC_038419]
	Rehmania virus 1	(ReV1)	[NC_040572]
Velarivirus	little cherry virus 1	(LChV-1)	[NC_001836]
	grapevine leafroll-associated virus 7	(GLRaV-7)	[NC_016436]
	Areca palm velarivirus 1	(APV1)	[NC_027121]
	Cordyline virus 1	(CoV-1)	[NC_038421]
	Cordyline Virus 2	(CoV-2)	[JQ599282]
	Cordyline virus 3	(Cov-3)	[JQ599283]
	Cordyline virus 4	(CoV-4)	[JQ599284]
Crinivirus	lettuce infectious yellows virus	(LIYV)	[NC_003618]
	sweet potato chlorotic stunt virus	(SPCSV)	[NC_004124]
	cucurbit yellow stunting disorder virus	(CYSDV)	[NC_004810]
	beet pseudo-yellows virus	(BPYV)	[NC_005210]
	strawberry pallidosis-associated virus	(SpaV)	[NC_005896]
	potato yellow vein virus	(PYVV)	[NC_006063]
	blackberry yellow vein-associated virus	(BYVBaV)	[NC_006963]
	tomato chlorosis virus	(ToCV)	[NC_007341]
	bean yellow disorder virus	(BnYDV)	[NC_010561]
	lettuce chlorosis virus	(LCV)	[NC_012910]
	tomato infectious chlorosis virus	(TICV)	[NC_013259]
	Diodia vein chlorosis virus	(DVCV)	[NC_038787]
	tetterwort vein chlorosis virus	(TEVCVO)	[NC_038789]
Ampelovirus	pineapple mealybug wilt associated virus-2	(PMWaV-2)	[AF283103]
	pineapple mealybug wilt associated virus-3	(PMWaV-3)	[DQ399259]
	grapevine leafroll-associated virus 3	(GLRaV-3)	[NC_004667]
	little cherry virus 2	(LChV-2)	[NC_005065]
	plum bark necrosis and stem pitting-associated virus	(PBNSPaV)	[NC_009992]
	pineapple mealybug wilt-associated virus 1	(PMWaV-1)	[NC_010178]
	grapevine leafroll-associated virus 4	(GVLRaV-4)	[NC_016416]
	grapevine leafroll associated virus 1	(GLRaV-1)	[NC_016509]
	blackberry vein banding associated virus	(BVBaV)	[NC_022072]
	pineapple mealybug wilt associated virus-2	(PMWaV-2)	[AF283103]
Unassigned	Actinidia virus 1	(AcV-1)	[KX857665]
